# Carotid plaque regression following 6-month statin therapy assessed by 3T cardiovascular magnetic resonance: comparison with ultrasound intima media thickness

**DOI:** 10.1186/1532-429X-13-37

**Published:** 2011-08-03

**Authors:** Raymond Q Migrino, Mark Bowers, Leanne Harmann, Robert Prost, John F LaDisa

**Affiliations:** 1 Department of Medicine, Marquette University, 1120 W. Wisconsin Avenue, Wilwaukee, WI 53233, USA; 2 Radiology Department, Medical College of Wisconsin, 8701 Watertown Plank Road | Milwaukee, WI 53226, USA; 3 Cardiology Department, Phoenix Veterans Affairs Health Care System, 650 E. Indian School Rd., Phoenix, AZ 85012-1892, USA; 4 Biomedical Engineering Department, Marquette University, 1120 W. Wisconsin Avenue, Wilwaukee, WI 53233, USA

## Abstract

**Background:**

Cardiovascular magnetic resonance (CMR) allows volumetric carotid plaque measurement that has advantage over 2-dimensional ultrasound (US) intima-media thickness (IMT) in evaluating treatment response. We tested the hypothesis that 6-month statin treatment in patients with carotid plaque will lead to plaque regression when measured by 3 Tesla CMR but not by IMT.

**Methods:**

Twenty-six subjects (67 ± 2 years, 7 females) with known carotid plaque (> 1.1 mm) and coronary or cerebrovascular atherosclerotic disease underwent 3T CMR (T1, T2, proton density and time of flight sequences) and US at baseline and following 6 months of statin therapy (6 had initiation, 7 had increase and 13 had maintenance of statin dosing). CMR plaque volume (PV) was measured in the region 12 mm below and up to 12 mm above carotid flow divider using software. Mean posterior IMT in the same region was measured. Baseline and 6-month CMR PV and US IMT were compared. Change in lipid rich/necrotic core (LR/NC) and calcification plaque components from CMR were related to change in PV.

**Results:**

Low-density lipoprotein cholesterol decreased (86 ± 6 to 74 ± 4 mg/dL, p = 0.046). CMR PV decreased 5.8 ± 2% (1036 ± 59 to 976 ± 65 mm^3^, p = 0.018). Mean IMT was unchanged (1.12 ± 0.06 vs. 1.14 ± 0.06 mm, p = NS). Patients with initiation or increase of statins had -8.8 ± 2.8% PV change (p = 0.001) while patients with maintenance of statin dosing had -2.7 ± 3% change in PV (p = NS). There was circumferential heterogeneity in CMR plaque thickness with greatest thickness in the posterior carotid artery, in the region opposite the flow divider. Similarly there was circumferential regional difference in *change *of plaque thickness with significant plaque regression in the anterior carotid region in region of the flow divider. Change in LR/NC (R = 0.62, p = 0.006) and calcification (R = 0.45, p = 0.03) correlated with PV change.

**Conclusions:**

Six month statin therapy in patients with carotid plaque led to reduced plaque volume by 3T CMR, but ultrasound posterior IMT did not show any change. The heterogeneous spatial distribution of plaque and regional differences in magnitude of plaque regression may explain the difference in findings and support volumetric measurement of plaque. 3T CMR has potential advantage over ultrasound IMT to assess treatment response in individuals and may allow reduced sample size, duration and cost of clinical trials of plaque regression.

## Background

Carotid atherosclerotic disease is a major risk factor for stroke [1] and a marker of systemic plaque burden [2,3]. Carotid intima-media thickness (IMT) by B-mode ultrasound is the current standard for carotid evaluation as well as clinical trial endpoint. However, in multiple trials using 3-hydroxy-3-methylglutaryl coenzyme A reductase inhibitors or statins, reduction in plaque burden as measured by IMT is modest and slow [2-5] as compared to improvement in clinical outcomes [6-8].

Unlike ultrasound IMT, cardiovascular magnetic resonance (CMR) of the carotid artery allows volumetric measurement of plaque. Carotid CMR provides reproducible measurements, excellent spatial resolution and has been validated with histology [9-14]. A recent study using CMR quantified regional heterogeneity in plaque distribution related to regional wall shear stress [15], implying the need for full volumetric measurement.

Animal and human studies show that intensive lipid lowering can lead to rapid change in plaque burden, decreasing as early as 9 weeks [16,17]. Using 1.5 Tesla (T) CMR, Lee and coworkers [18] demonstrated 3.1% reduction in carotid plaque over 3 months of statin treatment in 24 statin-naïve acute coronary syndrome patients. Similarly Corti and colleagues showed plaque regression in 18 patients treated with statins for 12 months [19,20]. Unlike ultrasound IMT, carotid CMR has been shown to detect plaque regression at an earlier time point. 3T CMR, in contrast to 1.5T, provides improved signal to noise ratio that can be used to improve image quality or shorten acquisition time [21]. Having a reliable noninvasive method for early detection of plaque regression is important for assessment of individual response to treatment as well as to reduce sample size and cost of clinical trials of novel therapies. To our knowledge, carotid plaque regression using 3T CMR has not been compared with ultrasound IMT in patients treated with statins. We aim to test the hypothesis that carotid plaque volume measured by 3T CMR will decrease following 6 month statin treatment in patients with carotid atherosclerosis and that plaque regression will not be detected using ultrasound IMT.

## Methods

### Study Subjects

Twenty-six consecutive volunteers (67 ± 2 years, 7 female) with atherosclerotic disease (coronary artery disease [n = 15, 58%] or cerebrovascular disease [n = 11, 42%]) and ≥1.1 mm carotid plaque thickness (IMT) on screening B-mode ultrasound from 1 institution were prospectively enrolled. Informed consent was obtained from all subjects and the study was approved by the local Institutional Review Board. The subjects underwent same-day CMR and ultrasound of the carotid arteries at baseline and following 6 months of statin treatment. Six had initiation of statin treatment, 7 had intensification of statin treatment (these 13 subjects comprise the statin increase group) while 13 subjects had maintenance of statin regimen (statin maintain group) within 2 weeks of baseline studies, as prescribed by their care providers (Table 1). The decision on statin dosing was based on the judgment of the primary neurologist or cardiologist taking care of the patient, with a few patients deemed to be at very high risk for a repeat neurologic or ischemic event treated with high statin doses (e.g. recent embolic stroke in subjects 3 and 6 in Table 1). Subject 1 was a participant in an initial clinical trial wherein she was started on atorvastatin 80 mg/day following the diagnosis of moderate/severe carotid artery disease. Baseline and 6-month lipid profile and high sensitivity C-reactive proteins were obtained using standard laboratory methods.

**Table 1:** Patient Characteristics and Statin Treatment

Treatment Groups	Age (year)/Gender	Baseline Treatment (mg)	Treatment During Study (mg)
**A. Statin Increase: Initiation**			
1	79/F	none	atorvastatin 80
2	72/M	none	simvastatin 20
3	68/F	none	atorvastatin 40
4	56/F	none	pravastatin 40
5	86/M	none	atorvastatin 20
6	46/M	none	rosuvastatin 40
**Statin Increase**			
**7**	58/M	atorvastatin 10	atorvastatin 80
8	72/F	simvastatin 10	atorvastatin 80
9	87/M	lovastatin 40	atorvastatin 80
10	61/M	simvastatin 40	simvastatin 80
11	73/M	pravastatin 80	atorvastatin 80
12	65/M	atorvastatin 10	atorvastatin 40
13	66/M	simvastatin 20	simvastatin 40
**B. Statin Maintain**			
14	62/M	atorvastatin 40	simvastatin 20
15	61/M	atorvastatin 10	simvastatin 20
16	71/M	lovastatin 40	lovastatin 40
17	64/M	simvastatin 80	simvastatin 80
18	57/M	simvastatin 40 (noncompliant with prescribed treatment)	simvastatin 40 (noncompliant with prescribed treatment)
19	71/M	simvastatin 5	simvastatin 5
20	63/M	Ezetimibe 10/simvastatin 20	Ezetimibe 10/simvastatin 20
21	56/F	simvastatin 10	simvastatin10
22	65/M	atorvastatin 10	atorvastatin 10
23	82/M	simvastatin 20	simvastatin 20
24	60/M	simvastatin 20	simvastatin 20
25	81/F	atorvastatin 10	atorvastatin 10
26	61/M	pravastatin 40	pravastatin 40

### Magnetic Resonance Imaging

**Figure 1:**
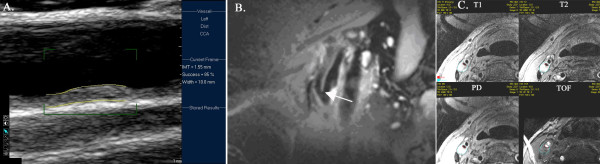
**Ultrasound and CMR images**. A. B-mode ultrasound of the common carotid artery demonstrating plaque. Automated software detection is used to measure mean posterior IMT. B. Oblique sagittal spin echo of the carotid artery showing the reference flow divider (white arrow). C. Multicontrast CMR T1/T2/proton density spin echo and time of flight gradient echo images of the same axial slice. Luminal/adventitial borders are outlined to measure plaque area/volume.

CMR was performed using a 3T General Electric scanner (Chalfont St. Giles, UK) and 4-channel carotid surface coil (Clinical MR Solutions, Brookfield, WI) at baseline and 6 months. A custom-built foam head/neck holder reduced head mobility to replicate positioning together with the built-in midline laser localizer. An oblique sagittal spin echo image of the index carotid artery (the artery with the most plaque burden; analyses were performed only on index arteries) was used to determine flow-divider position; images were obtained ± 12 mm from the flow divider (Figure 1). Multicontrast imaging was performed using axial T1 (repetition time-TR, 800 ms, echo time-TE, minimum, inversion time-TI 650 ms), T2 (TR-4000 ms, TE-50 ms, TI-250 ms), proton-density (PD, TR-4000 ms, TE-minimum, TI-250 ms) spin echo and time-of-flight (TOF, TR-20 ms, TE-minimum) gradient echo sequences (2 mm slice thickness, spatial resolution 0.31-0.62 × 0.31-0.62 × 2 mm), similar to previous studies [11].

### CMR Plaque Volume Quantification

T1/T2/PD/TOF CMR images of the index artery were simultaneously displayed on the monitor as previously described [15]. Using software (MR-Plaqueview, VP Diagnostics, Seattle WA), a single reader blind to subject identity and study order measured plaque volume (PV). The adventitial/luminal borders were traced and the area in between multiplied by slice thickness to obtain PV; the sum for all 12 slices comprised CMR PV. Tracing was initially performed from T1-weighted images but software then allowed simultaneous viewing of luminal and adventitial tracings in all 4 contrast weightings (T1, T2, PD and TOF) in 4 contiguous panels on the computer screen. The tracings could then be further adjusted to best fit the lumen and adventitia borders based on all 4 contrast images. The software also automatically delineates plaque components based on contrast characteristics using an automatic classifier (morphology-enhanced probabilistic plaque segmentation algorithm) [10,11,22]. Plaque composition from 1.5T CMR images derived using the software has been validated to correlate with histology while results on 1.5T correlated highly with 3T imaging [10,11]. The volume of lipid-rich necrotic core (LR/NC) and calcification components were measured and compared between baseline and 6-months and the changes were correlated with change of plaque volume.

From 52 carotid scans, 13 were randomly chosen and reanalyzed in blinded fashion to obtain intraobserver variability. The coefficient of variability was 5.3%, comparable to published intraobserver values for both 1.5T and 3T CMR of 6.4-7.7% [13,14].

For circumferential plaque distribution, the carotid luminal and adventitial tracings were imported into MATLAB, as previously published [15]. The artery was divided into 6 non-overlapping circumferential regions with 360 (or 0) degree arbitrarily selected as the most medial point in a standard axial slice with angular designation proceeding anterolaterally. The software provided measurements for the maximum thickness of each circumferential region, and the average for all slices calculated and compared. The investigators were meticulous in ensuring as similar as methodologically possible head positioning between baseline and 6-month scans. This was accomplished by the specialized design of the carotid coil that had custom-built head rest and neck support that minimized variations in three-dimensional head positioning when the center of the nose and philtrum of the subject are perfectly aligned with the midline laser localizer. Because of the absence of accepted convention for designating circumferential regions, the most medial portion of the carotid artery (an easily identifiable point) was arbitrarily chosen as corresponding to 360 (or 0) degree.

### Ultrasound

Carotid B-mode ultrasound was performed using a Philips iE33 ultrasound (Philips Medical Systems, Andover, MD) and L11-3 linear transducer by a qualified and experienced sonographer. Measurements were performed at the R wave of the electrocardiogram. The common carotid artery, bifurcation and internal carotid arteries were imaged on long axis at baseline and 6 months. To replicate baseline view, a Meijer Arc (Meyer Medical Ultrasound, The Netherlands) was used. The head was rotated 45° to the contralateral side with the transducer placed 45° from midline. The sonographer had visual access to baseline images (separate screen) while obtaining 6-month images to ensure consistency and replication of baseline views.

IMT was measured using automated methods (QLab IMT Quantification Software, Philips Medical Systems, Andover, MD, Figure 1). Edge-detection algorithm determined the intima-media interfaces and provided graphic display superimposed on the image. Manual editing was performed if needed but was kept to a minimum. The average of the mean posterior IMT of the common carotid/bifurcation/internal carotid artery in 10-mm segments was used for analysis, similar to ENHANCE trial method [23].

### Statistical Analyses

Data are expressed as mean ± standard error of mean. Baseline and 6-month variables were compared using paired Student's t-test. Circumferential plaque thickness was analyzed using repeated measures analysis of variance with region and time as covariates (Sigmastat 3.5, Systat Software, Richmond CA); pairwise comparison used Holm-Sidak method. Intraobserver variability was determined using coefficient of variability (standard deviation of difference in PV between two measurements over mean of PV*100%), similar to prior studies [24]. Correlation analysis was performed using Pearson correlation. Significant p-value was < 0.05.

## Results

Following 6 month statin treatment, there was an overall reduction of 14% and 9% decrease in LDL and total cholesterol, respectively, with greater decrease in the statin increase group compared to the statin maintain group (Table 2). At 6 months, mean LDL cholesterol was 74 ± 4 mg/dL. There was no change in HDL cholesterol or C-reactive protein.

**Table 2:** Lipid and high sensitivity C-reactive protein values

Group	Laboratory Value	Baseline	6-months	p-value
Overall	Total cholesterol	161 ± 7	146 ± 5	**0.04**

	LDL	86 ± 6	74 ± 4	**0.046**

	HDL	44 ± 2	44 ± 2	NS

	Triglyceride	149 ± 17	138 ± 13	NS

	hsCRP	2.2 ± 0.5	2 ± 0.4	NS

Statin Increase	Total cholesterol	175 ± 10	142 ± 6	**0.002**

	LDL	96 ± 8	73 ± 7	**0.014**

	HDL	42 ± 2	44 ± 2	NS

	Triglyceride	161 ± 22	123 ± 10	NS

	hsCRP	2.3 ± 0.7	1.8 ± 0.5	NS

Statin Maintain	Total cholesterol	146 ± 8	129 ± 8	NS

	LDL	77 ± 9	62 ± 6	NS

	HDL	45 ± 4	43 ± 3	NS

	Triglyceride	136 ± 27	120 ± 24	NS

	hsCRP	2.2 ± 0.8	2.3 ± 0.8	NS

HDL-high density lipoprotein cholesterol, hsCRP-high sensitivity C-reactive protein, LDL-low density lipoprotein cholesterol

**Figure 2:**
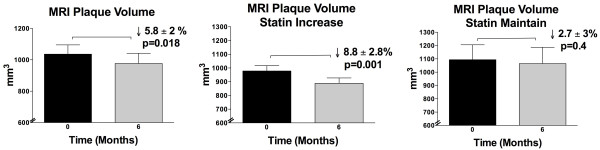
**CMR Plaque volume**. A. All subjects, B. Statin increase group, C. Statin maintain group. Overall there was reduction in CMR PV. Reduction was significant only in the statin increase group.

**Figure 3:**
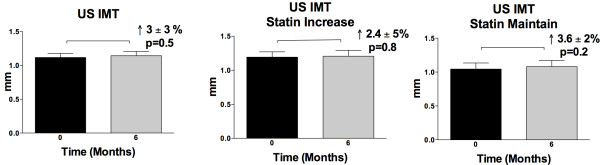
**Ultrasound IMT**. A. All subjects, B. statin increase group, C. statin maintain group. There was no significant change from baseline-6 months.

There was a 5.8 ± 2% reduction in CMR plaque volume overall (baseline versus 6-month: 1036 ± 59 versus 976 ± 65 mm^3^, p = 0.018, Figure 2). The statin increase group had 8.8 ± 2.8% reduction in plaque volume (979 ± 38 versus 889 ± 39 mm^3^, p = 0.001) while the statin maintain group had a nonsignificant change of -2.7 ± 3% in plaque volume (1094 ± 112 versus 1064 ± 123 mm^3^, p = NS, Figure 2). In contrast, ultrasound IMT showed a nonsignificant +3 ± 3% change overall (1.12 ± 0.06 versus 1.14 ± 0.06 mm, p = NS); the same was true for both statin increase (1.19 ± 0.08 versus 1.21 ± 0.09 mm, p = NS) and maintain groups (1.04 ± 0.09 versus 1.08 ± 0.09 mm, p = NS, Figure 3).

**Figure 4:**
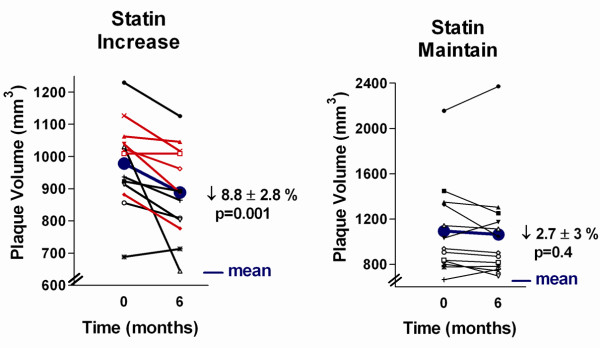
**CMR plaque volume reduction**. A. Statin increase group (red represents statin naïve subjects, black represents statin intensification subjects), B. statin maintain group. Blue line represents mean for the group.

In the statin increase group, 11/13 (85%) had reduced plaque volume while 9/13 (69%) had reduced plaque volume in the statin maintain group (Figure 4).

Using software, plaque composition was analyzed. There was a correlation between percent plaque volume change from baseline to 6-months and percent change in LR/NC volume (R = 0.62, p = 0.006) with more modest correlation with percent change in calcification volume (R = 0.45, p = 0.03). Overall, average percent LR/NC volume change was -2.3 ± 10% (baseline versus 6-month volume: 155.4 ± 22 versus 150.6 ± 26 mm^3^, p = NS), with statin increase group changing -18.1 ± 14% (154.7 ± 26 versus 124.3 ± 24 mm^3^, p = NS) and statin maintain group changing 13.4 ± 12% (156.2 ± 37 versus 177 ± 47 mm^3^, p = NS). Overall average percent calcification volume change was -13.1 ± 12% (90.3 ± 15 versus 86.5 ± 23 mm^3^, p = NS).

**Figure 5:**
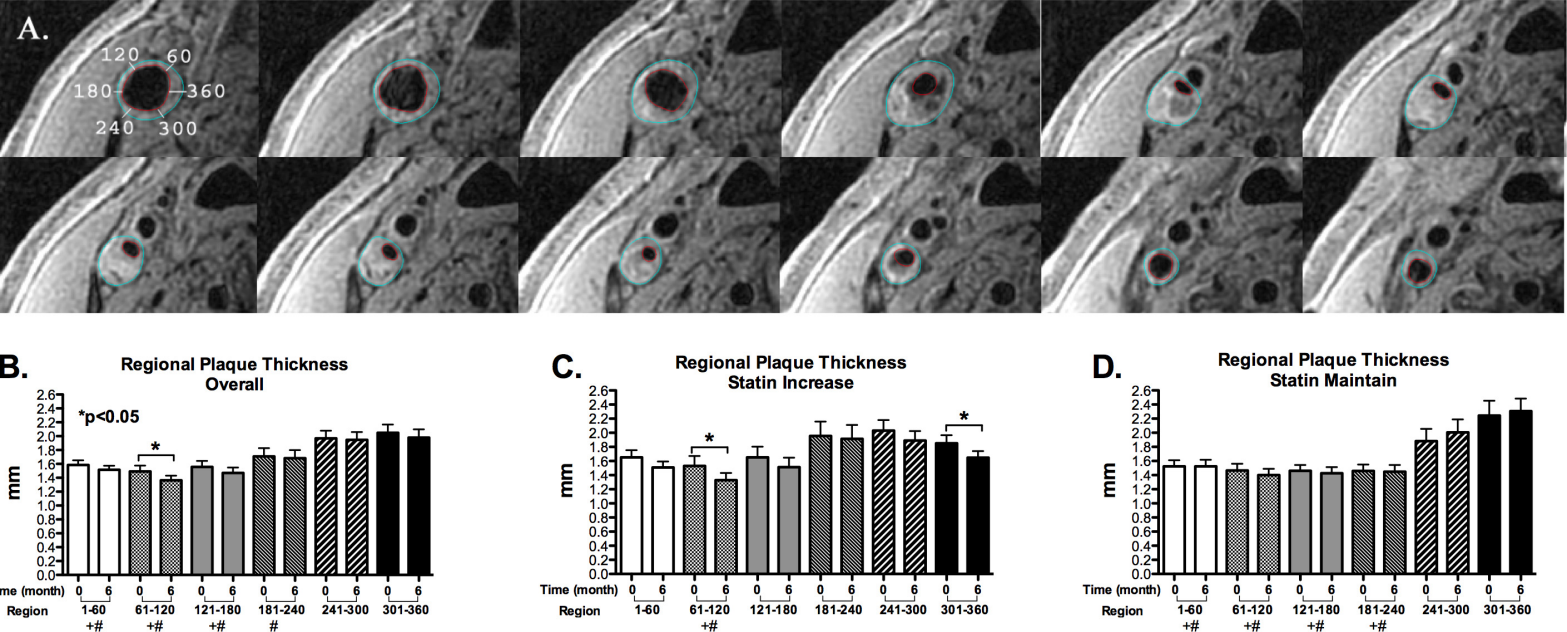
**Circumferential plaque distribution**. A. T1 images showing circumferential heterogeneity in plaque thickness. Angular designation is shown in the first image, with the most medial point arbitrarily designated as 360 degrees. B-D. Regional plaque thickness on CMR at baseline and 6 months. Note significant regional variation in plaque thickness (+p < 0.05 versus 241-300 degrees at both 0 and 6 month periods, #p < 0.05 versus 301-360 degrees at both 0 and 6 month periods) with greater plaque thickness at 241-300 and 301-360 degree regions. In all subjects, significant change in plaque thickness was only seen in 61-120 degree region (B); in statin increase patients, significant change was seen in 61-120 and 301-360 degree regions (C). There was no significant regional change in thickness in statin maintain patients (D).

There was circumferential heterogeneity in CMR plaque thickness with greatest thickness in the posterior carotid artery, in the region opposite the flow divider (241-360 degrees) (Figure 5). Similarly there was circumferential regional difference in *change *of plaque thickness with significant plaque regression in the anterior carotid region in region of the flow divider (61-120 degrees) overall (Figure 5B), and in the same region as well as 301-360 degrees in the statin increase group (Figure 5C). There was significant reduction in plaque thickness in statin increase group (-7.1 ± 3.6%, p = 0.016) but not in the statin maintain group (2.4 ± 5%, p = NS). There was no significant reduction in CMR plaque thickness when both groups are combined (-2.4 ± 2.5%, p = NS).

## Discussion

We report the following novel findings: in patients with coronary/cerebrovascular disease and carotid atherosclerosis, 6 month statin therapy led to reduced plaque volume using 3T CMR. However, there was no reduction in ultrasound posterior IMT. CMR plaque regression was greater in subjects who had initiation or increase of statins. There was circumferential regional heterogeneity in plaque thickness and regional heterogeneity in plaque regression. Important implications of this study include demonstrating the ability to measure plaque regression on a short term basis using high field CMR that will be relevant for following individual patients, or potentially reducing the cost, sample size and duration of clinical trials through the use of a reliable outcome marker. The study points to the need for volumetric measurement of carotid plaque.

### Measures of Plaque Regression

Carotid IMT as a measure of plaque burden is strongly associated with increased risk for development of coronary heart disease in both men and women [25,26]. Lipid lowering therapy using statins have consistently demonstrated ~25% reduction in major cardiovascular events in patients with stable atherosclerotic disease [7,8,27]. In high risk patients with acute coronary syndrome, initiation of statin therapy reduced major adverse cardiovascular events as early as 4-16 weeks [28,29]. In contrast, carotid IMT shows relatively slow reduction in plaque burden even after 1-2 years of high dose statin treatment [2-5,30] while some studies even showed absence of plaque regression by IMT, especially in lower risk subjects [30-33]. The ENHANCE trial showed that despite 56% reduction in LDL cholesterol, 2-year treatment with ezetimibe/simvastatin failed to show plaque regression by IMT [23]. The recent ARBITER 6-HALTS study showed lack of IMT regression in ezetimibe/statin treated patients after 14 months despite 21% reduction in LDL cholesterol, while showing IMT regression in niacin/statin treated patients [34]. Dissociation between significant reductions in cardiovascular events despite lack of carotid IMT regression with rosuvastatin in the JUPITER trial [32,35] points to the potential weakness of change in carotid IMT as a surrogate marker for clinical cardiovascular events [36].

Animal and human studies clearly demonstrate that aggressive lipid lowering can lead to measurable reduction in plaque volume in a short time period of as early as 6-9 weeks [16,17] suggesting that the temporal gap between plaque burden regression and clinical benefit may not be as large as carotid IMT studies suggest.

Carotid CMR provides distinct advantages over carotid IMT in the evaluation of carotid plaque burden. In contrast to the 2-dimensional carotid IMT measurement, CMR allows full volumetric measurement of plaque burden. CMR has been utilized to assess carotid plaque regression following statin treatment using 1.5 Tesla systems [9,18-20]. Carotid CMR is associated with high spatial resolution, excellent interstudy, interobserver and intraobserver variability and has been directly validated with histology [9-14,18]. Corti and coworkers [19,20] demonstrated measurable reduction in carotid plaque after 12 months of statin treatment. In patients with known coronary artery disease who were statin naïve, Lee and coworkers showed that aggressive lipid lowering to LDL cholesterol levels of ~70 mg/dL (levels comparable with the current study) with various statins was associated with measurable reduction in carotid plaque (-3.1%) as early as 3 months of treatment using 1.5 Tesla carotid CMR, and this was associated with parallel improvement in brachial endothelial function [18]. Our study using 3T CMR showed results consistent with their findings. We demonstrate 5.8% PV reduction in statin-treated atherosclerotic patients leading to LDL cholesterol levels ~74 mg/dL. Unlike the previous study, however, only 6/26 subjects were statin-naïve (7 patients had intensified dosing and 13 had dose maintenance). Furthermore, our cohort's baseline LDL was 86 versus 112 mg/dL for the prior study. A unique aspect of our study is demonstrating the gradient of plaque regression in established atherosclerotic patients with intensified versus stable dosing. The reduction of PV in the statin-increase group was seen in 11 of 13 subjects (with 1 patient without change in PV). This finding is consistent with ASAP and ASAP extension studies [5,37] where it was demonstrated that patients treated with atorvastatin 80 mg/day had IMT reduction over 2 years versus progression in those treated with simvastatin 40 mg/day. After 2 years, those taking atorvastatin was maintained on the treatment while those taking simvastatin was switched to atorvastatin 80 mg/day. The group maintained on atorvastatin showed no further reduction in IMT while the simvastatin group who had intensified treatment showed reduction in IMT; at 4 years, there was no difference in IMT between the two groups.

A novel contribution of this study is the direct comparison of carotid plaque change using 3T CMR versus ultrasound IMT. It is important to note that CMR PV (a 3-dimensional measurement, in mm^3^, and that includes adventitia) is not directly comparable to IMT measurement derived from 2-dimensional images (in mm and excludes adventitia). So the important comparison is not the *magnitude *but rather the *direction *of change between the two measurements. Whereas CMR PV decreased by 5.8% at 6 months, there was a non-significant change of +3% in IMT. Our results demonstrate that change of plaque burden using IMT is not concordant with volumetric change assessed by CMR.

### Change in Plaque Composition

There was significant correlation in plaque volume regression and reduction in lipid rich/necrotic plaque volume, and a more modest correlation with reduction in plaque calcification. This suggests that plaque remodeling involves parallel reduction in gross volume as well as plaque components. Importantly, the changes in lipid rich/necrotic plaque and calcification volumes did not reach statistical significance, unlike plaque volume, suggesting that using plaque component change as an endpoint in therapeutic trials will require greater sample size than plaque volume change. Our results are consistent with the analysis of Saam, et al. [38] where measurement error was 1.9 times for lipid rich/necrotic core assessment than for plaque volume, hence requiring a larger sample size to be able to detect similar degree of change induced by a therapeutic intervention.

### Regional Heterogeneity in Plaque Burden

The difference between CMR PV and IMT change may lie in an important observation of circumferential heterogeneity in plaque distribution, a finding described by others [39]. There is more plaque in the carotid bifurcation, in the region opposite the flow divider (241-360 degree region) [15]. Posterior IMT, a region routinely used to measure plaque in clinical trials [23], captures the region with highest plaque burden. However, following statin treatment, we observed non-uniform circumferential plaque regression, with significant reduction only in the region of the flow divider (61-120 degrees), located anteriorly in an area not captured by posterior IMT measurement, and in some (301-360 degrees) but not all posterior regions. The inability of ultrasound to fully capture regional plaque burden change may explain the limitation of IMT to reliably follow up short-term plaque regression.

Our data on regional plaque thickness change on 26 subjects (Figure 5B) remain generally consistent with initial data on 8 subjects (5 statin increase and 3 statin maintain) that we previously reported [15]. With more subjects, however, we now demonstrate circumferential regional variability in plaque thickness change from baseline to 6 months in statin increase subjects (Figure 5C) and no significant change in plaque thickness in statin maintain subjects. This demonstrates the potential peril of using maximum plaque thickness in serial evaluation of plaque regression as even regions with greatest plaque thickness and whose posterior locations make them ideal for ultrasound IMT studies (241-300 and 301-360 degrees) show variation in change in plaque thickness. This again highlights the value and superiority of volumetric assessment of plaque. Of note in statin increase subjects, as shown in Figure 5C, there was significant reduction in plaque thickness from 0 to 6 months at 61-120 degrees, a region of relative low plaque burden, as well as 301-360 degrees, a region of relative high plaque burden, suggesting that the relationship between regional plaque remodeling and local hemodynamic forces (e.g. wall shear stress) may be more complex than we initially reported [15], and deserve further investigation. Our data on regional plaque thickness change are not directly comparable to the analysis performed by Corti, et al. [19] where they showed significant difference in carotid maximum plaque thickness but not in minimum thickness at baseline and 12 months of simvastatin treatment, since their measurements did not include circumferential regional determination of plaque thickness, hence regions of maximum and minimum thickness may not be exactly the same at 0 and 12 months.

If our results are validated in a larger series, there are important clinical implications. Our study reinforces the previous observations [18,24] that CMR can reliably detect change in plaque burden within a short time period (6 months), but which ultrasound IMT is not able to do. For an individual patient, this provides a novel method of assessing response to treatment that is measurable in the short term, allowing early titration of treatment. From a research standpoint, the findings point to the limitations of ultrasound IMT as an endpoint to assess therapeutic efficacy. Carotid CMR might allow earlier detection of treatment efficacy of novel treatments, and because of the excellent interstudy, interobserver and intraobserver variability profile [9,18], might require a smaller sample size to detect treatment effects, potentially leading to cost-effective clinical trials.

### Limitations

There are several important study limitations. The sample size is small and the study needs to be validated in a larger series of patients. The import of our findings should therefore be construed as hypothesis-generating and not conclusive. Despite the small size, however, the results are consistent with results obtained by prior investigators using 1.5T CMR with similar sample size [18,24]. Statins are generally believed to have similar class effect and the clinical efficacy is related to degree of LDL cholesterol lowering rather than to the specific type of statin used in multiple randomized clinical trials [40]. Despite this, however, another important study limitation is the variable statin dosing and regimen. The baseline differences in treatment (including some being statin naïve and some on statins) limit data interpretation. Although meticulous care was expended to replicate head positioning, subtle variation in head and carotid position can affect the measurements and may be minimized but not totally avoided.

## Conclusions

Six month statin therapy in patients with carotid plaque led to reduced plaque volume as measured by 3T CMR, whereas ultrasound posterior IMT did not show any change. The heterogeneous spatial distribution of plaque and regional differences in magnitude of plaque regression may explain the difference in findings and support volumetric measurement of plaque. 3T CMR has potential advantage over ultrasound IMT to assess treatment response in individuals and may allow reduced sample size, duration and cost of clinical trials of plaque regression.

## Competing interests

The authors declare that they have no competing interests.

## Authors' contributions

RQM participated in the conception, design and implementation of the study, data analysis and interpretation, manuscript preparation, MB participated in the implementation of the study, data analysis and interpretation and manuscript preparation, LH participated in the implementation of the study, data analysis and manuscript preparation, RP participated in the implementation of the study and manuscript preparation, JLD participated in the implementation of the study, data analysis and interpretation and manuscript preparation. All authors read and approved the final manuscript.
